# Addition of Ceftriaxone and Amikacin to a Ciprofloxacin plus Metronidazole Regimen for Preventing Infectious Complications of Transrectal Ultrasound-Guided Prostate Biopsy: A Randomized Controlled Trial

**DOI:** 10.1155/2017/4635386

**Published:** 2017-01-10

**Authors:** Mohammad-Hossein Izadpanahi, Kia Nouri-Mahdavi, Seyed Mahmood Majidi, Mohammad-Hatef Khorrami, Farshid Alizadeh, Mehrdad Mohammadi-Sichani

**Affiliations:** Department of Urology, Isfahan University of Medical Sciences, Isfahan, Iran

## Abstract

*Background.* The objective of this study was to evaluate the efficacy of adding single doses of ceftriaxone and amikacin to a ciprofloxacin plus metronidazole regimen on the reduction of infectious complications following transrectal ultrasound-guided prostate biopsy (TRUS Bx).* Materials and Methods.* Four hundred and fifty patients who were candidates for TRUS Bx were divided into two groups of 225 each. The control group received ciprofloxacin 500 mg orally every 12 hours together with metronidazole 500 mg orally every 8 hours from the day prior to the procedure until the fifth postoperative day. In the second group, single doses of ceftriaxone 1 g by intravenous infusion and amikacin 5 mg/kg intramuscularly were administered 30–60 minutes before TRUS Bx in addition to the oral antimicrobials described for group 1. The incidence of infection was compared between the groups.* Results.* The incidence of infectious complications in the intervention group was significantly lower than that in the control group (4.6% versus 0.9%, *p* = 0.017).* Conclusion.* The addition of single doses of intramuscular amikacin and intravenously infused ceftriaxone to our prophylactic regimen of ciprofloxacin plus metronidazole resulted in a statistically significant reduction of infectious complications following TRUS Bx.

## 1. Introduction

Prostate cancer is one of the most common cancers and the third cause of mortality in men. Transrectal ultrasound-guided prostate biopsy (TRUS Bx) is a standard tool for the diagnosis of prostate cancer. This procedure, however, may be associated with complications such as pain, hematuria, hemospermia, damage to the urethra, and urinary tract infection. Infectious complications are most important, as they can cause prostatitis and urosepsis, which can be life-threatening. The incidence of infectious complications after TRUS Bx is estimated between 1 and 7%. The figure has increased over the past ten years, however [1]. Unlike other lower urinary tract procedures, antimicrobial prophylaxis is recommended for all patients undergoing prostate biopsy, irrespective of risk factors. The 2014 updated latest AUA best practice policy recommended antibiotics for prostate biopsy include fluoroquinolones; first-, second-, and third-generation cephalosporins; aminoglycosides; trimethoprim-sulfamethoxazole; and aztreonam [2]. Despite the widespread use of antibiotics for TRUS Bx, infection following the procedure is still common. This may be due to the increasing bacterial resistance to fluoroquinolones. Infectious complications caused by anaerobes are also on the rise. Therefore, combined use of antibiotics for infection prophylaxis may have compelling justification. The purpose of this study was to investigate the efficacy of adding combined intramuscular amikacin and intravenous ceftriaxone to oral ciprofloxacin plus metronidazole for further reduction of infectious complications following TRUS Bx.

## 2. Materials and Methods

This study is a randomized clinical trial conducted from June 2010 to February 2013 at our institution on patients who were referred for prostate biopsy. Informed consent was obtained from all men who were enrolled in this study. Patients with evidence of urinary tract infection or tumor, urolithiasis, sexually transmitted disease, or urethral stricture were deemed ineligible. Patients who were lost to follow-up and men who did not comply with the antimicrobial regimen were excluded from data analysis. Simple random sampling was performed and using randomized block design, 450 patients who fulfilled the inclusion criteria were selected and were assigned to one of two groups of 225 each.

In the first group (control group), one ciprofloxacin 500 mg tablet was given every 12 hours together with metronidazole 500 mg orally three times daily from the day prior to the procedure until the fifth postoperative day. In the second group (intervention group), single doses of ceftriaxone 1 g by intravenous infusion and amikacin 5 mg/kg intramuscularly were administered 30–60 minutes before TRUS Bx in addition to the oral antimicrobials described for group 1.

All patients received liquids for dinner the night before the biopsy and used one bisacodyl suppository at 9 pm.

Systematic 12-core TRUS Bx was performed by a single urologist. Local anesthesia was achieved using intrarectal 2% lidocaine gel (up to 20 mL) as well as periprostatic 2% lidocaine injection bilaterally. All patients received written instructions to immediately present to our urology clinic or emergency room in case of new lower urinary tract symptoms (LUTS), fever, chills, or severe bleeding.

All patients with fever ≥38°C and one or more LUTS were considered to have an infectious complication and were hospitalized. Blood and urine cultures were performed.

At the end of the study, the two groups were compared using independent *t*-test and chi-square test (SPSS 22).

## 3. Results

This study included 450 patients undergoing prostate biopsy. Nine patients in the intervention group and 6 patients in the control group were excluded owing to noncompliance with follow-up, and the final analysis was carried out on 435 patients.

**Figure 1:**
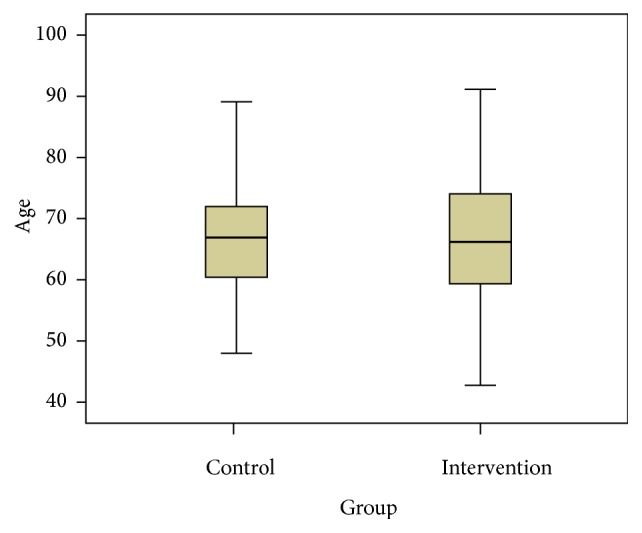
Median, range, and 25th and 75th percentile for age of the two groups.

The mean patient age was 66.5 ± 9.1 years. The mean ages of the control and intervention groups were 66.8 ± 8.9 and 66.3 ± 9.3 years (Figure 1), respectively, with no statistically significant difference between the two groups (*p* = 0.56).

**Figure 2:**
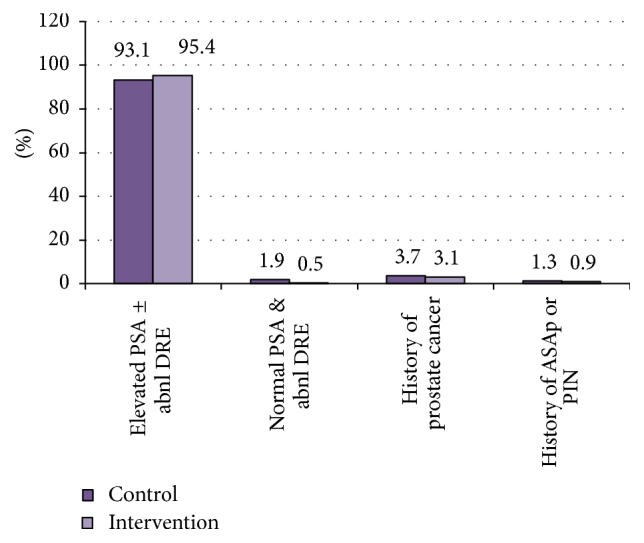
Frequency of indications for prostate biopsy in the two groups.

In both groups the most common indication for prostate biopsy was elevated PSA (associated with either normal or abnormal DRE) in 201 and 209 patients (93.1% versus 95.4%) of control and intervention groups, respectively, and Fisher's exact test indicated no significant difference between the two groups (*p* = 0.94).  Figure 2 shows the frequency of indications for biopsy in the two groups.

The mean serum PSA levels in the control and intervention groups were 21.2 ± 34.8 (range 1.5–413) and 16.7 ± 21 (range 1–161) ng/mL, respectively, and there was no statistically significant difference between the two groups (*p* = 0.11).

The percentage of free/total PSA was available for 71 patients in the control group and 60 in the intervention group. The mean % free/total PSA in the intervention and control groups were 16.6 ± 7.4 (6–35) and 15.2 ± 6.3 (6–32), respectively, and no statistically significant difference was observed between the two groups (*p* = 0.11).

The mean transitional zone volume in 166 patients in the control group and 117 patients in the intervention group was 22.7 ± 16.6 (range 4–123) cc and 23.7 ± 14.1 (range 5–76) cc, respectively, and no statistically significant difference was observed between the two groups (*p* = 0.59).The mean total prostatic volumes in control and intervention groups were 55.7 ± 41.4 (14–470 cc) and 59 ± 31.1 (15–190 cc), respectively, and no statistically significant difference was found between the two groups (*p* = 0.39). Additional data for both groups are shown in Table 1.

Of the 435 patients who underwent transrectal biopsy, 12 patients (2.8%) were hospitalized due to infection, 10 of whom (4.6%) were from the control group and 2 patients (0.9%) from the intervention group, with the difference being statistically significant (*p* = 0.017). The average time of biopsy to hospitalization in all patients was 6.25 ± 4.3 (1–14) days. Times to hospitalization was 5.6 ± 4.3 and 9.5 ± 3.5 days for intervention and control groups, respectively, but this difference between the two groups was not statistically significant (*p* = 0.26). The mean length of hospitalization was 4.3 ± 1.6 (2–8) days for all patients and 4.5 ± 1.7 and 3.5 ± 0.71 days in the intervention and control groups, respectively, with no statistically significant difference between the two groups (*p* = 0.45).

Of the 12 patients hospitalized with the diagnosis of infection, urine culture results were positive in 5 patients, 3 of whom were from the control group and 2 from the intervention group (30% versus 100%) but this difference was not statistically significant (*p* = 0.15). Blood culture was positive in 4 patients, 3 of whom were from the control group and 1 in the intervention group (30% versus 50%) but the difference was not statistically significant (*p* = 0.99). The results are shown in Table 2. Backward conditional logistic regression analysis on the obtained data indicated that parenteral antibiotics (ceftriaxone plus amikacin) had a significant effect on reducing the risk of infection after biopsy. Individual data of patients hospitalized with infection are shown in Table 3.

## 4. Discussion

Infection following TRUS Bx is one of the most common and significant complications in patients undergoing prostate biopsy and can entail hospitalization and additional cost. In fact most postbiopsy hospitalizations result from infectious causes. Antimicrobial prophylaxis is recommended for all patients undergoing prostate biopsy, irrespective of risk factors. Several prophylaxis protocols have been suggested in order to reduce the incidence of infectious complications but none has demonstrated unequivocal superiority.

A 2011 Cochrane review on prophylaxis for TRUS prostate biopsy demonstrated that antibiotics reduced bacteriuria, bacteremia, fever, urinary tract infection (UTI), and hospitalization compared to placebo or no treatment [3]. There was no definitive evidence demonstrating superiority of longer course or multiple doses compared to a shorter course or single any-dose protocols.

The frequency of complications following TRUS Bx has been recently noted to be increasing. Carignan et al. attributed the increasing incidence of post-TRUS Bx infections in their center to ciprofloxacin resistance [4]. Independent risk factors for post-TRUS Bx infection in that study were diabetes, hospitalization during the preceding month, and chronic obstructive pulmonary disease.

The use of fluoroquinolones in the previous 6 months before biopsy was found by Steensels et al. to be a risk factor for faecal carriage of fluoroquinolone-resistant* E. coli* strains and for infectious complications after TRUS Bx [5]. They recommend that the universal administration of fluoroquinolones should be reconsidered.

Adibi et al. observed an increase in hospital admissions related to infection after transrectal ultrasound-guided biopsy which led them to initiate an augmented regimen of one dose of intramuscular gentamicin before biopsy in addition to 3 days of ciprofloxacin or Bactrim DS [6]. The rate of hospitalization due to postbiopsy infections dropped from 3.8% to 0.6%. This augmented antibiotic prophylaxis was also found to be highly cost-effective.

In Taylor et al.'s study, targeted prophylaxis using rectal swab cultures to identify fluoroquinolone-resistant bacteria was compared with standard empirical ciprofloxacin prophylaxis [7]. Fluoroquinolone-resistant organisms were found in 19.6% of patients who received targeted prophylaxis. A considerable decrease in the incidence of infectious complications after transrectal ultrasound-guided prostate biopsy caused by fluoroquinolone-resistant organisms was noted after targeted antimicrobial prophylaxis, which was associated with sizeable cost savings on cost-effectiveness analysis.

Kehinde et al. noted a steady increase in the incidence of septicemia after prostate biopsy in their unit when only oral ciprofloxacin was used prophylactically [8]. The addition of a single dose of intravenous amikacin (500 mg 30 minutes before the procedure) significantly reduced the incidence of septicemia after prostate biopsy.

In Hsieh et al.'s retrospective study, the addition of a single intramuscular gentamicin injection (80 mg) 30 minutes before the biopsy to an oral levofloxacin protocol reduced the infection-related complications from 6.2% to 0.74% [9].

The results of this study showed that the addition of parenteral antibiotics could lead to a significant reduction of infectious complications compared to oral antibiotic use. This is, as noted above, probably due to increasing resistance to fluoroquinolones.

Parenteral antimicrobial administration provides broad-spectrum antimicrobial activity as well as higher drug bioavailability, which in turn can lower the incidence of post-TRUS Bx infectious complications. It should be noted that antimicrobial agents vary in their prostate-permeability characteristics. Ciprofloxacin and amikacin, which were used in this study, are able to penetrate the fatty membrane, whereas cephalosporins are not.

Our results showed that intervention and control groups were not significantly different in terms of age, indication for biopsy, and PSA levels. Confounding factors are, therefore, most unlikely to have affected the incidence of infectious complications. Multivariate analysis of the data showed that the incidence of infection after prostate biopsy in patients receiving parenteral ceftriaxone and amikacin in addition to oral antibiotic prophylaxis was significantly lower than that in the group treated with oral antibiotics.

Our study has several important limitations. Over-the-counter availability of some antimicrobials in our country is clearly associated with the spectrum of multiresistant germs present and may affect the generalizability of our work. Although all patients received written instructions to immediately refer to our urology clinic or emergency room in case of new lower urinary tract symptoms (LUTS), fever, chills, or severe bleeding, there may have been instances of infectious complications where patients might have received treatment elsewhere. We may have thus underestimated the true incidence of postbiopsy infectious complications. In addition, the routine use of our proposed prophylactic regimen may lead to the selection of resistant strains, further complicating the treatment of postbiopsy infections. Also, we are unable to account for comorbidities (especially diabetes) in our cohort, since this is an important cofactor for developing prostatitis following biopsy. Finally, the patients have to incur the additional expense of parenteral antimicrobials. Further studies focusing on cost-benefit analysis are thus warranted.

## 5. Conclusion

The addition of single doses of intramuscular amikacin and intravenously infused ceftriaxone to our prophylactic regimen of ciprofloxacin and metronidazole resulted in a statistically significant reduction of infectious complications following TRUS Bx.

## Figures and Tables

**Table 1:** Distribution of basic and prostate variables in both groups.

Variables		Groups		
		Control	Intervention	*p*
Number		216	219	—
Age (year)		66.8 ± 8.9	66.2 ± 9.3	0.56
Indication for biopsy	Elevated PSA ± abnormal DRE	201 (93.1)	209 (95.4)	0.94
	Abnormal DRE + normal PSA	4 (1.9)	1 (0.5)	
	History of prostate cancer	8 (3.7)	7 (3.1)	
	History of ASAP or PIN	3 (1.3)	2 (0.9)	
PSA (ng/ml)		21.2 ± 34.8	16.7 ± 21	0.11
% fPSA/total PSA (N)		16.6 ± 7.4 (71)	15.2 ± 6.3 (60)	0.35
Transition zone volume (N)		22.7 ± 16.6 (166)	23.7 ± 14.1 (117)	0.59
Total prostatic volume (cc)		55.7 ± 41.4	59 ± 31.1	0.39

**Table 2:** Frequency distribution of the infection and the related symptoms of the two groups shown separately.

Variables		Groups		
		Control	Intervention	*p*
Infection *N* (%)	Yes	10 (4.6)	2 (0.9)	0.017
	No	206 (95.4)	217 (99.1)	
Time of biopsy-hospitalization		5.6 ± 7.3	9.5 ± 3.5	0.26
Hospitalization time (day)		4.5 ± 1.7	3.5 ± 0.71	0.45
Urine culture *N* (%)	Negative	7 (70)	0 (0)	0.15
	Positive	3 (30)	2 (100)	
Blood culture *N* (%)	Negative	7 (70)	1 (50)	0.99
	Positive	3 (30)	1 (50)	

**Table 3:** Characteristics of patients with infection after biopsy in control and intervention groups.

Groups	Patient	Age	Time biopsy- hosp.	Hosp. time	U/C	B/c
Control	1	65	14	2	Negative	Negative
	2	63	11	5	Negative	Negative
	3	60	3	5	Negative	Negative
	4	67	1	3	Positive	Negative
	5	74	6	3	Negative	Negative
	6	70	3	4	Positive	Negative
	7	48	8	5	Negative	Positive
	8	65	1	6	Negative	Negative
	9	60	4	4	Positive	Positive
	10	70	5	8	Negative	Positive

Intervention	11	78	12	4	Positive	Negative
	12	72	7	3	Positive	Positive
